# Accelerated Repurposing and Drug Development of Pulmonary Hypertension Therapies for COVID-19 Treatment Using an AI-Integrated Biosimulation Platform

**DOI:** 10.3390/molecules26071912

**Published:** 2021-03-29

**Authors:** Kaushik Chakravarty, Victor G. Antontsev, Maksim Khotimchenko, Nilesh Gupta, Aditya Jagarapu, Yogesh Bundey, Hypatia Hou, Neha Maharao, Jyotika Varshney

**Affiliations:** VeriSIM Life, 1 Sansome Street, Suite 3500, San Francisco, CA 94104, USA; kaushik.chakravarty@verisimlife.com (K.C.); victor.antontsev@verisimlife.com (V.G.A.); maksim.khot@verisimlife.com (M.K.); nilesh.gupta@verisimlife.com (N.G.); aditya@verisimlife.com (A.J.); yogesh.bundey@verisimlife.com (Y.B.); hypatia.hou@verisimlife.com (H.H.); info@verisimlife.com (N.M.)

**Keywords:** COVID-19, ACE inhibitors, calcium channel blockers, artificial intelligence, drug repurposing

## Abstract

The COVID-19 pandemic has reached over 100 million worldwide. Due to the multi-targeted nature of the virus, it is clear that drugs providing anti-COVID-19 effects need to be developed at an accelerated rate, and a combinatorial approach may stand to be more successful than a single drug therapy. Among several targets and pathways that are under investigation, the renin-angiotensin system (RAS) and specifically angiotensin-converting enzyme (ACE), and Ca^2+^-mediated SARS-CoV-2 cellular entry and replication are noteworthy. A combination of ACE inhibitors and calcium channel blockers (CCBs), a critical line of therapy for pulmonary hypertension, has shown therapeutic relevance in COVID-19 when investigated independently. To that end, we conducted in silico modeling using BIOiSIM, an AI-integrated mechanistic modeling platform by utilizing known preclinical in vitro and in vivo datasets to accurately simulate systemic therapy disposition and site-of-action penetration of the CCBs and ACEi compounds to tissues implicated in COVID-19 pathogenesis.

## 1. Introduction

The emerging pandemic of COVID-19 presents an extraordinary challenge in identifying effective drugs for prevention and cure [1,2]. Analysis of the cumulative surveillance data have shown progressively growing number of cases, which are now confirmed to be 100 million plus, as demonstrated by the World Health Organization emergency COVID19 informational dashboard [3]. In the current pandemic scenario, the average mortality rate was observed to be almost 3.0% [4]. Although the average mortality rate is declining, 7-day moving averages of daily incidence of COVID-19 cases indicate ongoing community transmission [5]. Outstanding preventive measures enforced in many countries and increased load on the health care system has provoked the highest economic impact in XXI century. Decrease of the weekly economic index in the US during the pandemic period could potentially reach 12%, which was never observed within the recent decades [6]. As prevention and containment of each COVID-19 outbreak is paramount in present situation, contingency measures with experimental therapeutics are being urgently investigated for the immediate unmet medical need.

There are several monotherapies that have been tested for COVID-19. Antiviral drug remdesevir was shown to be effective for the treatment of COVID-19 in adult patients and was officially approved by FDA in the United States [7]. The speed of the COVID 19 vaccine development was [8]. Despite some critical notes [9,10], a few anti-COVID-19 vaccines were approved, and public vaccination procedure have been started. At the same time, various pharmaceuticals compounds against coronavirus infection are still being tested and yet to demonstrate their significantly high efficacy rates over placebo as well as lower mortality. Despite the recent advances in antiviral therapeutic approaches, the current pipeline for drug interventions for COVID-19 consists largely of older antivirals, immunomodulatory agents, and traditional Chinese medicines (Table 1) [11]. Hence, a multitargeted combinatorial approach may have a greater potential to be a more successful therapy than a single drug target due to the multifactorial-polygenic infectious nature of the virus. In this critical scenario, development of a novel combination of antiviral medications is a promising approach and has the potential to be completed in the near future. Currently, the most effective way of the COVID-19 treatment is the use of the antibody drug cocktails such as casirivimab and imdevimab combination (REGN-COV2 or REGEN-COV2) [12,13]. Antibody drug cocktail-based treatments may have potential immunogenicity and hypersensitivity adverse effects in patients with COVID-19 [14], making the use of small molecule pharmaceutical potentially more beneficial.

There is a need for accelerated development of effective interventions as COVID-19 confirmed cases are increasing remarkably at a fast pace. While intensive research and clinical trials to address this critical unmet need are being developed to determine the efficacy of known drugs and identify potential therapeutic targets to develop new drugs for treating COVID-19, results to date on the drug efficacy are inconclusive and inconsistent, and safety profiles are unknown in the context of the disease [15]. Recently, a number of other drugs such as combinations of ACE inhibitors and CCBs were also considered for development [7]. Their anti-inflammatory, anti-fibrotic and vasodilatory roles have been well established in the pathophysiology of pulmonary hypertension (PH), a chronic health condition recognized as a high risk factor in severe COVID-19 disease [16]. ACE inhibitors and CCBs, used in the treatment for PH, have shown therapeutic efficacy in COVID-19 [17,18], when investigated separately. Hence, it can be postulated that CCBs in combination with key proteins such as ACE, pertaining to the renin–angiotensin signaling system (RAS) family, acting indirectly via ACE, can be potential targets to combat viral entry and replication and the post-infection proinflammatory responses known as the “cytokine storm” [19].

The growing knowledge regarding SARS-CoV-2 pathophysiology provides a significant number of potential drug targets, specifically focused on the virion structure and target tissues. SARS-COV-2 is a large (27–32 kb), enveloped, positive-stranded RNA virus. Viral capsid consists of four proteins: membrane protein, envelope protein, spike protein, and nucleocapsid protein [27]. The spike protein facilitates the entry of the virus into host cells [27], and it is a critical factor of viral host range and tissue tropism and is a major inducer of host immune response and disease severity [18,28,29]. Viral spike protein is involved in receptor binding and subsequent viral entry into the host cells [28]. At least initially, it binds to the cellular angiotensin-converting enzyme 2 receptor (ACE2), cellular receptor TMPRSS2, and the calcium channel post entry in nasal secretory goblet cells, lung type II pneumocytes, and gut absorptive enterocytes [30]. Subsequently, these proteins pertaining to the RAS can be considered as potential therapeutic targets. Moreover, angiotensin II type-I receptor blockers (ARBs), as well as thiazolidinediones and ibuprofen have been reported to increase the expression of ACE2, thereby increasing the risk of infection [31]. Therefore, among the components of RAS, ACE, a zinc-metallopeptidase converting Angiotensin (Ang) I to Ang II is considered as a most promising therapeutic target. Ang II mostly exerts its activity via a type 1 and type 2 angiotensin receptor maintaining blood pressure homeostasis, and anti-inflammatory response in addition to salt and fluid balance [32]. ACE2 generates Ang (1–7) from Ang II, and then Ang (1–7) after binding and activating the mitochondrial assembly 1 (MAS) receptor broadly, shifts the balance from vasoconstriction with Ang II to vasodilation, in particularly, in pulmonary vessels [33]. The role of this vasodilatory effect in the pathogenesis of COVID-19 is not studied yet, but some animal data suggest a beneficial effect in lung disorders in PH [33]. Additionally, ACE2 and Ang (1–7) have been found to be protective in several different lung injury models [33,34]. ACE inhibitors may potentially attenuate the COVID-19 associated “cytokine storm” by upregulating ACE2, which converts Ang II to Ang (1–7) and activate MAS receptors producing beneficial vasodilatory and anti-inflammatory effects that were shown to play a potential role regarding post-infection of COVID-19 [35].

The underlying mechanism of action of CCBs on SARS-CoV-2 needs further elucidation as the underlying mechanism is not fully understood. Several pathogenic viruses have been known to induce intracellular calcium influx by hijacking predominantly the voltage-gated Ca^2+^ channels (VGCC) facilitating viral entry, replication, and prolonged infection period [36,37]. In previous studies with Ebola and similar viruses CCBs displayed inhibition of the replication of viruses after entry [33,34,38]. Case fatality rates were markedly reduced due to the CCB treatment among patients that were infected with Severe Fever with Thrombocytopenia Syndrome Virus (SFTSV) [39]. Similarly to SFTSV patients, CCBs are postulated to interfere with SARS-CoV-2 replication after cellular entry. CCBs may act as host-signaling targeted compounds that reduce the rate of viral mutations and interferes with the replication process via modulation of virus-hijacked host cellular machinery, compared to antivirals which target many viral proteins [40]. As a result, this could be an important factor in the development of compounds against SARS-CoV-2 [40]. The putative mechanism of action of CCBs entails the interference of the intracellular calcium influx instigated by the virus and the blocking of calcium-dependent signaling pathways pivotal to viral replication. The transient receptor potential channel (TRP) is known to be associated with hypersensitivity induced by chemical or thermal stimuli. Infection of human bronchial epithelial cells by respiratory viruses including respiratory syncytial virus (RSV), measles virus (MV) and rhinovirus (RV) was found to increase the expression of TRP channels in human bronchial epithelial cells. The over-expression of TRP proteins provides a favorable environment for propagation of virus [17,41]. Additionally, a key consequence of viral pathogenesis is marked by a strong inflammatory response preceded by a set of sequentially activated signaling pathways such as increased intracellular calcium levels leading to mitochondrial dysfunction and eventually cellular apoptosis [42,43,44]. In prior clinical outcomes, CCBs attenuated markedly the proinflammatory response by modulating the intracellular calcium levels to homeostasis in patients and reduced death rates in septic animal models with the high systemic proinflammatory state [45,46]. Additionally, the global attenuation of proinflammatory cytokines and oxidative stress by CCBs was observed in hypertensive patients compared to baseline [47]. It can be hypothesized that CCBs, besides interfering with viral replication, may attenuate systemic inflammatory responses in patients to impart the clinical benefits synergistically with their antiviral efficacy. These therapies may be particularly relevant to COVID-19 given its association with an extended proinflammatory state in patients [19].

**Figure 1:**
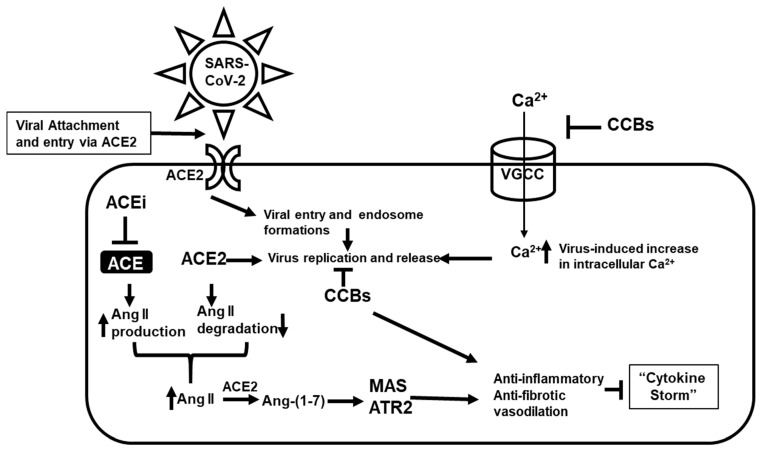
Potential model for the combinatorial actions of angiotensin-converting enzyme inhibitors (ACEi) and calcium channel blockers (CCBs) on SARS-CoV-2 infection, replication, and proinflammatory response. ACEi acting indirectly through ACE2 and the mitochondrial assembly 1 (MAS), ATR2 axis imparts an anti-inflammatory and anti-fibrotic response, while CCBs acts at various steps, restores intracellular Ca^2+^ flux, consequently inhibiting post-infection virus internalization and genome replication. Both ACEi and CCBs potentially can induce a synergistic anti-inflammatory and anti-fibrotic response in the attenuation of the “cytokine storm”.

Taken together, combinatorial approaches using ACE inhibitors, acting in an anti-inflammatory fashion, and CCBs countering the virus post-entry-stage, re-establishing Ca^2+^ homeostasis, and consequently down-regulating the proinflammatory signals, may impart synergistic outcomes in increasing the clinical efficacy via reducing viral load in patients compared to the individual drug treatments (Figure 1). While novel in concept, there is insufficient data on ACE/CCB combinatorial therapies available to verify whether these observations are translatable to humans, as no studies have evaluated the effects of combinations of RAS inhibitors and CCBs in COVID-19 clinical trials. To that end, we propose an in silico based computational approach for repurposing and predictions of optimal dosage and disposition of combination of ACEis and CCBs to potentiate therapeutic efficacies in clinical trials.

In the present study, the BIOiSIM was used for the computational prediction of ACE inhibitor and CCB drug dispositions in the context of tissues related to COVID-19 pathogenesis. Two CCB drugs namely Verapamil and Lacidipine belonging dihydropyridine and phenylalkylamine derivatives were selected as they cover interaction with both T- and L-types of calcium channel in cardiovascular system. Captopril, lisinopril, and captopril were chosen as the typical ACE inhibitors belonging to sulfhydryl- and dicarboxylate-containing agents showing variable interactions with ACE active centers. BIOiSIM is a dynamic, biology-driven platform that provides a scalable computational prediction of in vivo pharmacokinetic-pharmacodynamic (PK-PD) phenomena. A potential treatment strategy for COVID-19 will consist of a two-pronged approach; initially, minimizing the rate and extent of in vivo SARS-CoV-2 infection/replication and second, reducing the systemic inflammatory effect implicated in the “cytokine storm” post-infection. The aim of the study was to use the BIOiSIM platform to conduct in silico modeling of the various CCB/ACE inhibitor compounds with integrated experimental preclinical datasets and prediction of the drug disposition to tissues, which were shown to be a site of virus residence such as nasal epithelium, lungs, and intestine [48]. Investigation of the drug disposition in these tissues would help accelerate the development of the targeted anti-COVID-19 therapy.

## 2. Results

### 2.1. Sensitivity and Convergence Testing

**Figure 2:**
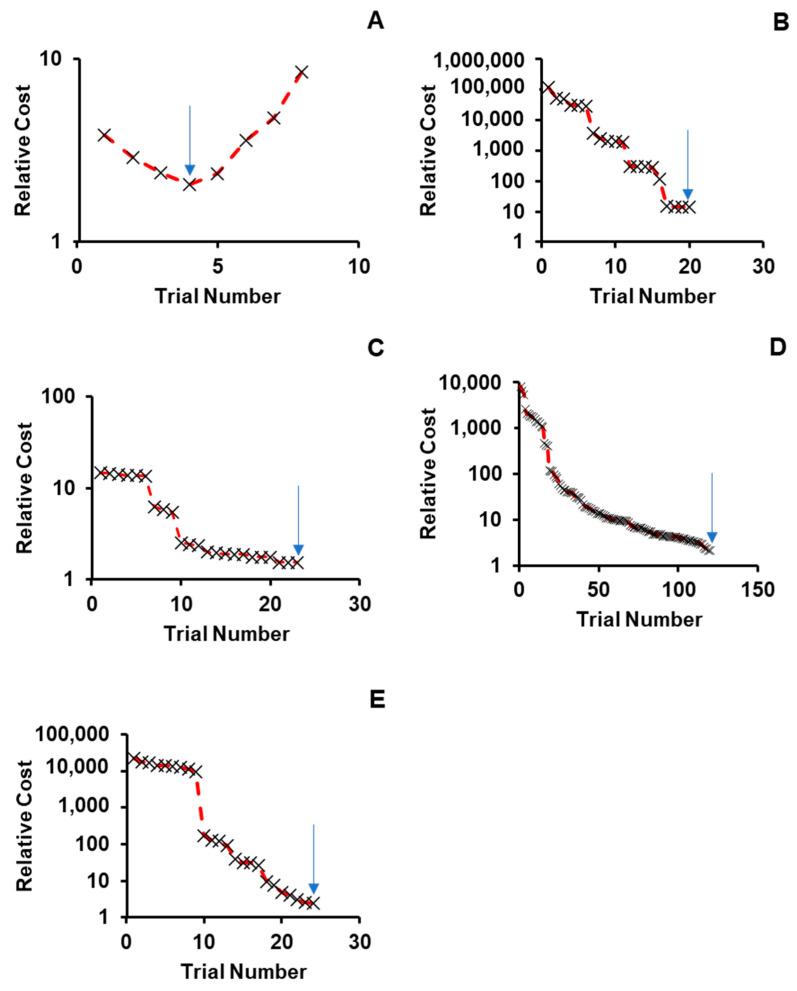
Convergence onto discrete parameter combinations during coarse optimization for (**A**) Verapamil, (**B**) Spirapril, (**C**) Lisinopril, (**D**) Lacidipine, and (**E**) Captopril. Relative cost on the y-axis is calculated as: Cost_relative = AAFECmax*AAFEtmax*AAFEAUC where AAFECmax, AAFEtmax, AAFEAUC—absolute average fold error for Cmax, Tmax and AUC predictions.

For cases where parameter values were not available experimentally, BIOiSIM’s optimization algorithms were utilized to obtain parameter values enabling simulation of drug disposition in both the plasma venous compartment as well as the COVID-19-associated sites-of-interest. Figure 2 highlights these outcomes by showing how the objective function (cost) varies over the trials that were tested during the coarse grid search phase of optimization. There is confidence in the optimized parameter values because of the clear absolute minima that can be visualized, looking at the convergence plots for Verapamil, Spirapril, Lisinopril, Lacidipine, and Captopril (convergence point highlighted with an arrow). The final optimized values are summarized in Table 2. Table 3 gives an overview of the experimental conditions in the in vivo datasets used for the study, which were replicated for the simulation outputs.

### 2.2. Simulation Accuracy

**Figure 3:**
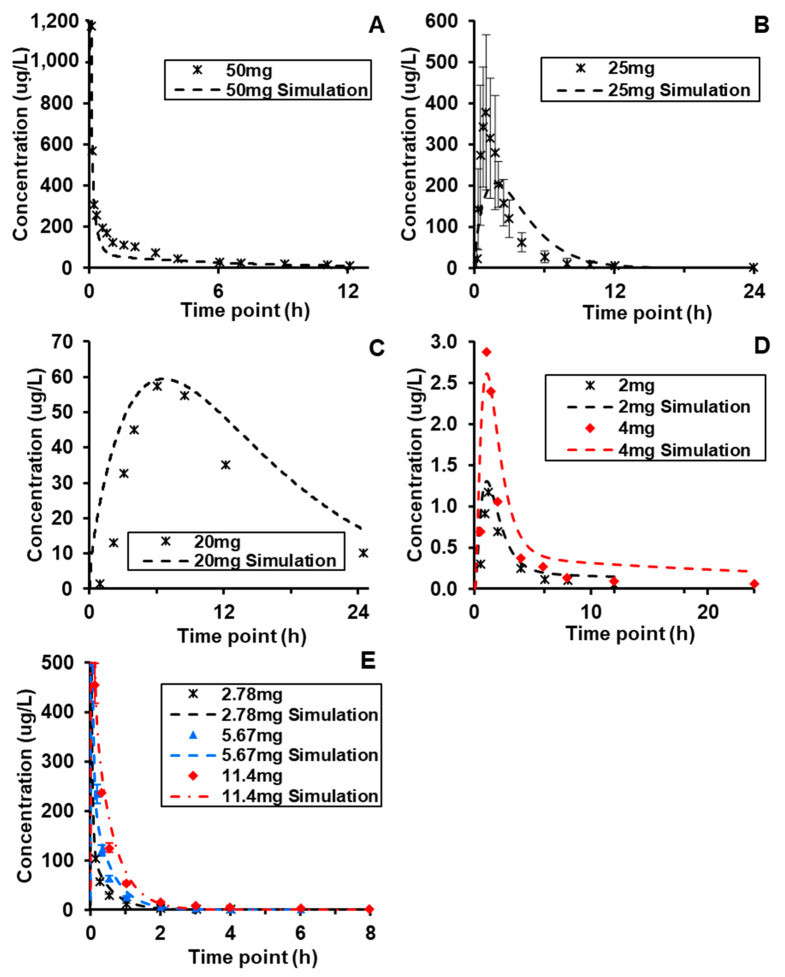
Dose and route-of-administration dependent prediction of compound plasma concentration for (**A**) Verapamil (i.v.), (**B**) Spirapril (p.o.), (**C**) Lisinopril (p.o.), (**D**) Lacidipine (p.o.), and (**E**) Captopril (i.v.). Dashed lines correspond to BIOiSIM simulation outputs. Error bars and individual data points were digitized from the original publications (49,51,55,56,58) and correspond to standard error/standard deviation, as presented in the original work.

BIOiSIM simulations of plasma concentration over time relative to experimental data are captured in Figure 3 [49,51,55,56,58]. The performance of the core model across the different compounds was assessed using a combination of AFE and AAFE values across the metrics generated from non-compartmental analysis and chi-squared hypothesis testing. Additionally, visual analysis of the simulation fits against the validation data was conducted to assess goodness-of-fit, and served as a key metric in assessing the platform performance in cases where error/variability data was not available from the original source. As highlighted in Table 4, the AAFE for T_max_, C_max_, and AUC0-t for all of the compounds—Lisinopril, Captopril, Spirapril, Lacidipine, and Verapamil—was less than 2, indicating that the simulation results were close to recapitulating the actual in vivo behavior. The average AFE values for the compound metrics (1.03, 0.92, and 1.08 for AUC_0–t_, C_max_, and T_max_, respectively) are within fold-error of ±0.1 and indicate minimal bias toward systematic over- or under-prediction of output values. Both IV and orally administered compounds were simulated with similar accuracy. Spirapril had slightly higher AAFE values for C_max_ and T_max_ (1.93, 1.75 respectively); however, this can be attributed to the higher variability in the in vivo datasets, as visualized in Figure 3 and confirmed with the chi-squared statistic (*p*-value < 0.001).

Visual analysis of the plots is also indicative of overall high accuracy, with the simulation outputs accurately captured the ascending and terminal phase that aligns with the expectation for IV and Oral routes-of-administration. For Lacidipine and Captopril, the two compounds where data was available for multiple doses, the maintained accuracy of simulation outputs (AAFE < 1.3 across all metrics) supports the model’s ability to accurately simulate clearance and absorption after oral administration of the compounds. The chi-squared metrics and associated *p*-values did not show statistically significant fits; as referenced in Table 2, the majority of publications reviewed for in vivo data did not contain timepoint-specific variability, and assumptions made regarding variability are likely too conservative and result in a failure to reject the null hypothesis of difference in the plots.

### 2.3. Simulating Distribution to Gut, Lung, and Nasal Epithelium

**Figure 4:**
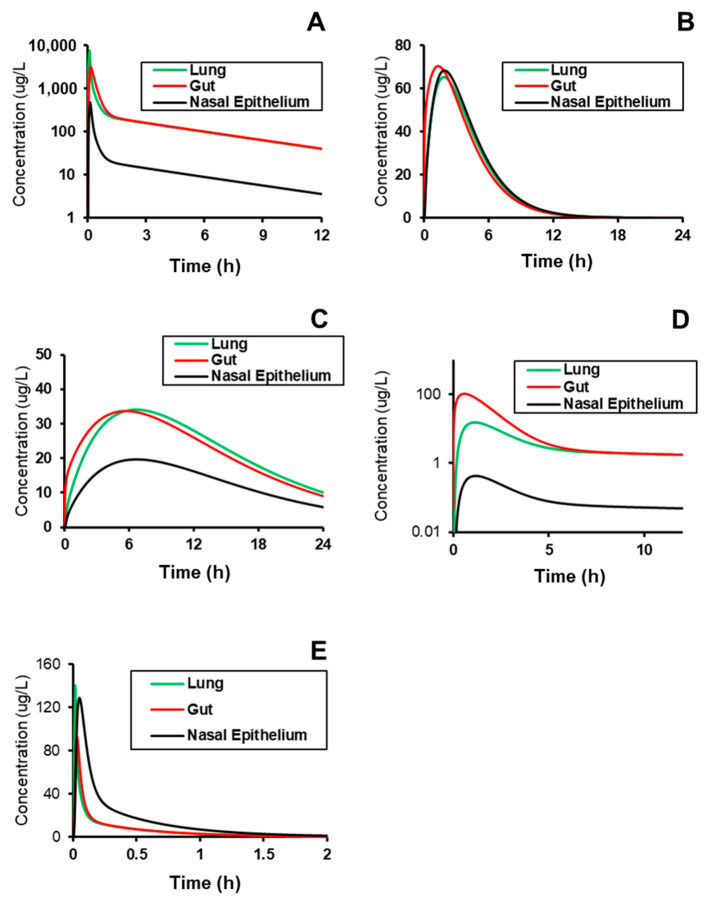
Prediction of the site-specific distribution of (**A**) Verapamil (i.v.), (**B**) Spirapril (p.o.), (**C**) Lisinopril (p.o.), (**D**) Lacidipine (p.o.), and (**E**) Captopril (i.v.) to tissues implicated in COVID-19 replication and pathogenesis. Subject conditions are described in Table 3.

After successfully validating the systemic plasma PK across compounds, predictive models generated outputs of expected tissue concentration profiles in the three COVID-implicated tissues—gut, lung, and nasal epithelium (Figure 4) with calculated metrics and conditions (Table 5). The conditions that were simulated matched those used during model validation, and were assumed to be in the range of typically prescribed dosing regimens. The simulation values showed that based on the assumed permeability of nasal tissue, the overall exposure of compound in the nasal epithelium (AUC0-t) is effectively the same as in the general tissue; however, the C_max_ in the nasal tissue is greater for compounds administered via IV infusion by a factor of 1.4 and 2 for Verapamil and Captopril, respectively. This behavior is likely explained by slower distribution of compound into tissues than it is noted for PO administration allowing two nasal compartments to noticeably equilibrate. Thus, it is more important to assume the permeability to the site-of-action may be an insignificant rate-limiting factor.

One interesting observation from these outcomes is that the effective lag time in T_max_ between the nasal epithelium and the other tissues is minimal, which may indicate that permeability to the site-of-action is an insignificant rate-limiting factor. Additionally, the relative C_max_ is <2-fold different at the nasal epithelium relative to other tissues, and indeed, the other tissues are also similar to plasma levels specifically for Spirapril, Lisinopril, and Captopril. These compounds may therefore be better candidates for rapid repurposing, given that their systemic plasma profiles are likely to have data around interaction with Ca^2+^ and ACE in PH patients, and those levels are directly correlated to those in tissue.

## 3. Discussion

The COVID-19 pandemic has been converted to a global crisis on an unprecedented scale. With synchronous and monumental efforts among researchers worldwide, several novel and old therapeutic treatments have been investigated and promoted without definitive or explicit protocols. Few therapeutic regimens may have developed at a risk; fortuitously, some demonstrate initial hope and potential efficacy. Albeit, current published results of exhaustive clinical trials are yet to be interpreted and evaluated for therapeutic impacts in patients. Several clinical studies that are focusing on the treatments of patients with COVID-19 have demonstrated an urgent unmet medical need to determine and establish the optimal treatment for therapeutic approaches currently being tested in a clinical context. To that end, we developed an in silico modeling approach in repurposing of CCBs and ACEi compounds, using an AI-integrated mechanistic modeling platform by utilizing known preclinical in vitro and in vivo datasets to accurately simulate systemic therapy disposition and site-of-action penetration of the CCBs and ACEi compounds to tissues implicated in COVID-19 pathogenesis. Numerous studies have been carried out so far confirming increased ACE expression in response to the ACEi administration. Several hypotheses exist about how increased tissue ACE2 expression may be protective rather than harmful during SARS-CoV-2 infection. For example, increased ACE2 expression may lead to enhanced sequestration of SARS-CoV-2. Moreover, ACEis’ lead to competition with Ang II for AT1R, resulting in increased Ang II to be processed by ACE2. This increases Ang (1–7) levels, which results in vasodilating and anti-fibrotic effects, providing crucial protection during coronavirus infections. Furthermore, increased binding of ACE2 to circulating Ang II could induce a conformational change resulting in less favorable binding of SARSCoV-2 to its receptor and decreased internalization of the virus when bound to ACE2 [59]. Subsequently, numerous clinical studies have shown no evidence for deleterious effects of ACEi in COVID-19 patient. In fact, discontinuing these life-saving medications potentially can have adverse effects in these groups of patients [60].

In the present study, we have demonstrated that representatives of two drug classes, namely ACE2 inhibitors and CCB, which were recently shown to provide healing effects in COVIS-19 patients [15,57], possess beneficial pharmacokinetic profile with good accumulation rate in lung and nasal epithelium tissues. Despite some recent publication confirm significant improvement of the COVID-19 patients’ condition due to monotherapy with these drugs or their combination, there are neither computation data no experimental results published confirming their suitable pharmacokinetics in the site of coronavirus potential residence i.e., lung, nala epithelium, and intestine. The simulation results presented here may serve as potential baselines for metrics designed to repurpose and prioritize compound candidates based on their PK disposition. The simulation of compound penetration to lung, gut, and nasal tissues was based on direct optimization of partition coefficients to simulate the systemic compound distribution and/or calculation using the Rodgers-Rowland method (Table 2). Thus, fine-tuned dosing optimization of drug exposure to these specific tissues could vary relative to actual clinical outcomes and the kinetic properties (e.g., IC50) of the compounds themselves. However, as the PK profile simulation accuracy is high the effective distribution and overall potency/penetration of these compounds at a certain dose can be assumed to be relatively accurate and may have utility for prioritizing compounds based on potential for reaching a desired site-of-action. Given the potential variability inherent in the methods for compound partitioning, further experimental validation in vitro or in vivo would be required to gain full confidence in the distribution simulated to these sites.

**Figure 5:**
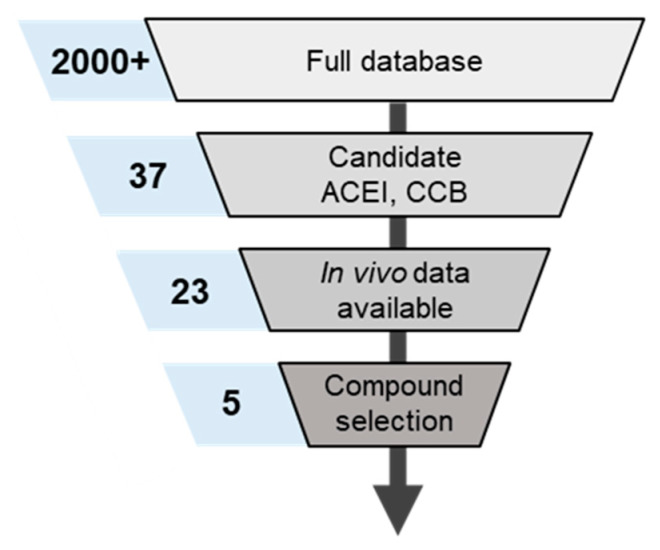
Metrics for candidate compound identification unitilizing internal database of small molecule PK. The numbers in blue boxes correspond to the number of compounds identified as acceptable during each stage of curatrion.

The approach taken serves as initial validation of computational tools for rapid repurposing of therapeutics with an established mechanism-of-action. Specifically, this lays the groundwork for a potential repurposing pipeline outside of COVID-19 and inclusive of unmet medical needs where existing drugs may target a pathway relevant to the condition. The general workflow of scanning a database of compounds, identifying accurately simulated datasets, and using proprietary optimization algorithms filled in knowledge gaps by optimizing missing experimental information within the bounds of physiological expectations enables the extension of simulation results beyond experimentally measured data and into case-specific prediction (Figure 5). Thus, centralized model-integrated databases well-curated datasets of in vivo compound PK are also critical for accelerated prioritization of candidates for emerging diseases. There is value in augmenting the data as selected candidates undergo further in vivo preclinical or clinical testing, specifically for building confidence in model simulation results.

**Figure 6:**
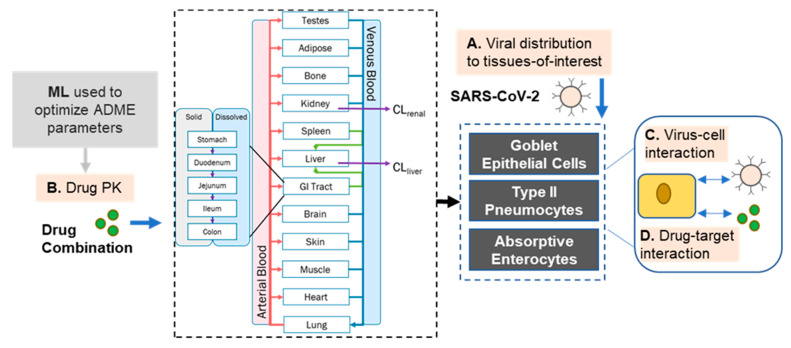
Diagram of modeling approach taken that extends beyond pharmacokinetic modeling of compound disposition and can explore more complex viral and combination effects.

Further extension and application of these results may enable greater insight into the potential therapeutic effects of ACEi and CCB combinations, as highlighted in Figure 6. One potential includes the development of PD models relating the extent of ACE inhibition to Ang II levels (postulated to play a role in the “cytokine storm” secondary to COVID-19) that could drive hypothesis-driven modeling and simulation of patient outcomes. Additionally, optimal dosing regimens of the compounds can be derived by exploring the expression levels of Ca^2+^ channels and ACE across the relevant tissues and extrapolating from in vitro studies of channel blocking and inhibition to assess the likelihood that a sufficient concentration of drug can reach a site-of-action to either minimize viral replication or reduce inflammatory effects. The levels of such endogenous mediators can be linked to the expected drug concentrations in plasma or active site to gain a deeper understanding of the interaction of drug with the active site components and their downstream pharmacological effects—integration of these PK-PD parameters with BIOiSIM platform can extend the prediction of drug disposition and safety and efficacy applicable to other drug therapies in healthy and diseased populations.

As more data is collected, and the pathophysiology of COVID-19 in healthy and diseased populations is understood, more customized prescriptions of specific CCB/ACEi combinations can be derived by looking at susceptibility to drug–drug interaction and variability in relevant gene expression levels between subjects. Overall, these results give additional confidence in the BIOiSIM platform’s ability to rapidly identify and simulate drug disposition for compounds that may be efficacious therapies for rapidly emerging, deadly conditions. Considering the universality ingrained in the core of the BIOiSIM platform, its predictive potential could be implemented across any modalities for repurposing and consequent treatment of infectious diseases as well as other clinical conditions.

## 4. Materials and Methods

### 4.1. Overview of BIOiSIM Platform

The core functionality of the in silico simulation platform described here was outlined in a recent work around modeling penetration of transdermal formulations to systemic circulation [47]. Briefly, the platform is comprised of a 16-organ model of compound PK, validated across an internal database of small molecule compounds. Auxiliary models (specific PK or PD) can be integrated into the centralized framework to expand the ability to make specific predictions of compound disposition. Artificial intelligence-Machine learning (AI-ML) algorithms are utilized to either optimize missing parameters in the case of insufficient experimental data, or as predictive solutions to train on existing in vivo/in vitro datasets. The software systems are hosted on Amazon Web Services (AWS) cloud, enable high throughput of parallelized PK simulations (PK simulator performance is ~0.08 s to simulate 1 hr of drug exposure at 0.36 s resolution) which is available in the VeriSIM Life customer portal.

The drug-dependent parameters used in the model are either experimentally determined or predicted/optimized using a combination of ML cost minimization algorithms; these include integrating iterative optimization algorithms with random walk methods (similar to Markov Chain/Monte Carlo) to converge on a global minimum. Details of the approach are discussed in our recent publication [61].

### 4.2. Modeling Compound Disposition

Computing the mass balance between the compartments follows the general form:

(1) $$V_{organ}\frac{dC_{organ}}{dt}=Q_{organ}(C_{blood,in}-\frac{C_{organ}}{K_{T:P,unbound}*f_{unbound,plasma}}*B:P)$$

For metabolizing and eliminating organs (e.g., liver, kidney) the general relationship in the model is defined as:

(2) $$V_{organ}\frac{dC_{organ}}{dt}=Q_{organ}\left(C_{blood,in}-\frac{C_{organ}}{K_{T:P,unbound}*f_{unbound,plasma}}*B:P-CL_{organ}*C_{organ}\right)$$

where tissue-dependent parameters are expressed as *V* (organ volume), *Q* (flow rate), *CL* (organ-level clearance), and *K* (unbound tissue:plasma partition coefficient); *B:P* represents whole blood to plasma partition ratio, and *C* is the drug concentration in the specific compartment.

This global approach has been previously described in our manuscript [61].

Given the identified relevant tissues for SARS-CoV-2 infection (nasal, lung, and intestine), a critical aspect of the approach was enabling simulation of compound disposition to those relevant sites-of-action. Generally, the tissues were assumed to be well-mixed; thus, distribution was characterized using a partition coefficient:

(3) $$K_{Tissue:Plasma}=\frac{C_{tissue}}{C_{plasma}}$$

where *C_tissue_* is concentration of the compound-of-interest in a particular organ/tissue, and *K_Tissue:Plasma_* is the drug/subject-specific partition coefficient in the tissue. The methodology utilized to determine the partition coefficient was derived from the simulation accuracy of the plasma-concentration curve. There are multiple different methodologies that have been utilized in an attempt to predict these partition coefficients accurately, including industry standards such as the Rodgers-Rowland and Schmitt equations. However, even these relationships are susceptible to significant variability [62,63,64]. These models predict compound partitioning as a function of logP (octanol-water partition coefficient), pKa, and plasma protein binding (fu,p); however, for specific groups of compounds, they are found to underperform in their predictive capabilities based on the simplifying assumptions used [62,63,64,65,66]. Therefore, we utilized the standard Rodgers-Rowland equation, optimization of the octanol-water partition coefficient, and direct optimization of an average Kp to increase the simulation accuracy for in vivo PK. Partition coefficient values were directly optimized for Captopril, Lacidipine, and Verapamil, while octanol-water partition values (logP) were optimized for Spirapril.

The nasal epithelium mechanism for compound distribution involved integration with a permeation across a barrier. Given the location of nasal goblet cells in vivo, compound into the epithelium was modeled with assumptions of 1D flux across the barrier derived from Fick’s Law. To approximate compound buildup in tissue and effective reduction of the concentration gradient without incorporating semi-infinite sink assumptions, the epithelium thickness was scaled down by a factor of two. To model penetration to the nasal epithelium, a model of small molecule penetration across a permeable barrier was utilized. The developed model was identified and adapted from a combination of approaches utilized for predicting penetration across the barrier:

(4) $$\frac{dC_{Na}}{dt}=Q_{Na}\left(C_{a}\;-\frac{BP*C_{Na}}{k_{pna}}\;\right)-P_{nasal}*A*\;\left(\frac{C_{Na}}{K_{pNa}}\;-C_{nasal\_epi}*f_{nasal}\;\right)$$

(5) $$\frac{dC_{nasal\_epi}}{dt}=P_{nasal}A_{surf}\left(\frac{C_{Na}}{K_{p,Na}}\;-C_{nasal\_epi}*f_{nasal}\;\right)$$

where *Na* refers to the nasal tissue compartment, *C_nasal_epi_* the concentration in the nasal epithelium, *f_nasal_* the fraction of compound bound to the tissue, *P_nasa_*_l_ the permeability across the barrier, and *A* the area across which compound diffusion can occur. Physiological parameters used in the model were aggregated from a diversity of sources [65,66,67]. For the purpose of these simulations, permeability was assumed to be 0.000101 cm/s for all of the compounds studied, and the partition coefficient into the nasal tissue was assumed to be equivalent to that of lung tissue, as reported in Table 2 [68,69,70].

### 4.3. Rapid Repurposing Workflow

#### 4.3.1. Test Dataset

An internal database largest proprietary curated database consisting of structure-related data for > 1 M compounds, >3700+ unique in vivo plasma concentration-time validation datasets from public and proprietary sources (signed data sharing partnerships) representing ~2000 unique compounds and 83 different subject populations (different species, gender, strain, sub-strain).

The dataset was compiled through a combination of automated data scraping and manual data curation, resulting in the development of over 41 coded/automated consistency checks that detect outliers and corrupt data coming from either published literature on FDA compounds or from ongoing data partnerships. This innovative data curation approach was applied to CCB/ACE data curation, import and validation.

Screening through the internal database of compounds, comprising >2000 small molecules PK parameters & in vivo datasets identified five compounds that targeted the pathway-of-interest and had sufficient validation data available for confident & rapid simulation of outcomes. To establish the combinatorial effect of ACEis/CCBs combinations on COVID-19 infection, an accurate prediction of drug concentration at a site-of-action is required. Figure 2 highlights the approach taken to identify the 5 drugs (3 ACEis, 2 CCBs) that were candidates for the study as a result of the available in vivo clinical data and preclinical PK parameters that were already established. to validate the ability to predict plasma concentration accurately (as the standard metric for PK).

#### 4.3.2. Statistics and Tools

The statistical methodologies utilized for the data analysis have been detailed in a previous work [61]. Briefly, in vivo plasma concentration datasets and associated error bars, when available, were manually digitized from source publications using “WebPlotDigitizer” version 4.2.34. Model development and validation were done using the in-house platform in Python with Cython integration; matplotlib (v2.0.2) and Numpy (v1.14.2) were auxiliary packages used in simulation deployment and analysis. Model validation and analysis of model goodness-of-fit/accuracy were conducted using three quantitative metrics: absolute average fold error (*AAFE*), average fold-error (*AFE*), and chi-squared statistic (Χ2) with associated *p*-value (null hypothesis defined as no difference in predicted vs. experimental measurements). Sensitivity of the model was evaluated using convergence plots generated during optimization of the parameters. Non-compartmental calculations were utilized to compare the accuracy of the simulations to the experimental data; *AAFE* and *AFE* were utilized to evaluate the accuracy of the PK outputs AUC_0–t_, *C_max_*, and *t_max_* using the general equations:

(6) $$AFE=Average\;fold\;error=10^{\frac{1}{n}\overset{n}{\underset{i=1}{\sum }}\log\left(\frac{predicted_{i}}{observed_{i}}\right)}$$

(7) $$AAFE=Absolute\;average\;fold\;error=10^{\frac{1}{n}\overset{n}{\underset{i=1}{\sum }}\left|\log\left(\frac{predicted_{i}}{observed_{i}}\right)\right|}$$

where *n* is the total number of compounds used in the analysis and *Predicted_i_*/*Observed_i_* correspond to predicted and observed values of PK parameters, respectively. *χ*^2^ was calculated using the relationship:

(8) $$\chi^{2}=\frac{1}{n}\overset{n}{\underset{i=1}{\sum }}{\left(\frac{predicted_{i}-observed_{i}}{observed_{error,\;i}}\right)}^{2}\;$$

where observed error is the standard error/deviation in the measurements in the individual experimental data timepoints, obtained by digitizing the error bars from the respective publications. Optimization convergence was driven and measured by AAFE of AUC, *C_max_*, and *t_max_*. Statistical calculations of *AFE*, *AAFE*, and visual plot analysis were done in GraphPad Prism version 8.4.1 (GraphPad Software, San Diego, CA, USA) and Microsoft Excel (2016).

#### 4.3.3. Subjects

Species-specific parameters used in the simulations were adapted from literature sources and included parameters such as organ flow rates, composition, volumes, and protein levels for the physiological compartments in the BIOiSIM model [71,72,73,74,75]. Parameters for the model of small molecule diffusion across the nasal tissue and epithelium were obtained from a thorough literature review [65,66,76,77].

## 5. Conclusions

As cumulative datasets are collected, and the pathophysiology of COVID-19 in healthy and diseased populations is understood, more customized prescriptions of specific CCB/ACEi combinations can be derived by looking at susceptibility to drug–drug interaction and the associated variability in relevant gene expression levels between subjects. The findings of the present study confirm the potential use of a combinatorial approach of CCB and ACEi as therapeutic agents against COVID-19 infection due to their favorable tissue distribution with sufficient level of accumulation in the gut, lung, and airway epithelium. These organs have been demonstrated to be sites of virus infection, residence, and replication. Therefore, the combinatorial therapy using these drug classes can provide therapeutic efficacy against COVD-19. Overall, these results evaluate a new paradigm in using AI/ML-driven computational modeling for repurposing and accelerating the drug development process for swiftly identifying and simulating drug disposition for compounds that may be effective therapies for rapidly emerging, deadly clinical conditions.

## Figures and Tables

**Table 1:** Select compounds currently in clinical trials for treating COVID-19.

Compound Name	Development	Overview	Source
Remdesivir	Gilead, Approved by FDA	Antiviral, host factor-targeted. RNA-dependent/RNA polymerase-targeted	[20]
APN01	APEIRON Biologics, Phase I	Pilot trial ongoing in China	[21]
Brilacidin	Innovation Pharmaceuticals, Phase II	Defensin mimetic drug candidate. Has shown antibacterial, anti-inflammatory, and immunomodulatory properties in several clinical trials	[22]
Hydroxychloroquine	Repurposed, Rejected	Host factor-targeted. Antimalarial drug that affects endosomal function and blocks autophagosome-lysosome fusion	[23]
Azithromycin	Repurposed	Host factor-targeted. Broad-spectrum antibiotic, blocks autophagosome clearance in human cells	[23]
Camostat	Repurposed	Host factor-targeted. TMPRSS2 inhibitor	[24]
Nafamostat	Repurposed	Host factor-targeted. TMPRSS2 inhibitor	[25]
Favipiravir	Repurposed, Approved in Russia, Japan	Host factor-targeted. RNA-dependent/RNA polymerase-targeted	[26]

**Table 2:** Classification of identified ACEi, CCB compounds, and utilized physicochemical and pharmacokinetic (PK) parameters values obtained from literature, default value approximation, or machine learning (ML) optimization.

Drug Name	Class	ka	LogP	pKa	Fu,p	B:P	Clearance (L/h/kg)	Kp_lung_	Kp_gut_
Lisinopril	ACEi	0.17 *	−1.115 [49]	3.17 (acid), 10.21 (base) [49]	0.99 [49]	0.71 *	0.072 [49]	0.57	0.50
Captopril	ACEi	N/A	0.34 [50]	4.01 (acid), −1.2 [49]	0.73 [49]	0.45 *	0.72 [49]	0.15 *	0.15 *
Spirapril	ACEi	0.53 [51]	0 *	3.62 (acid), 5.2 (base) [49]	0.314678 *	0.74 **	0.43 [51]	0.21 *	0.16 *
Lacidipine	CCB	1.7843 *	5.51 [52]	19.47 (acid), −6.4 (base) [49]	0.05 [53]	0.70 *	1.23 [49]	11.72 *	11.72 *
Verapamil	CCB	N/A	3.795 [54]	9.68 (base) [49]	0.064 [55]	0.88 [36,54]	0.84 [49,54,55]	3.69 *	3.69 *

*—optimized values, **—default values.

**Table 3:** Background on datasets used for systemic plasma-venous compartment disposition simulation and optimization of missing PK parameters.

Drug Name	Formulation	Experimental Setup	Reference
Lisinopril	20 mg, oral dose	20 mg of Lisinopril was given orally for 10 consecutive days. 8 subjects in the study.	[56]
Captopril	2.78 mg, 5.67 mg, 11.4 mg, IV dose	1 mL of intravenous injection at three different dosage levels was administered to 7 healthy subjects.	[57]
Spirapril	25 mg, oral dose	25 mg spirapril p.o. prepared by dissolving 25 mg of lyophilized spirapril in 50 mL tap water was given to the subjects. 16 subjects.	[51]
Lacidipine	2 mg, 4 mg, oral dose	Single dose of 2 mg and 4 mg of Lacidipine was administered. The study has a total of 24 subjects (12 male, 12 female)	[58]
Verapamil	50 mg, IV dose	5 subjects received 5 mg verapamil dissolved in 30 mL of saline infused over 5 min.	[55]

**Table 4:** Comparison of plasma venous PK metrics between model simulation outputs and experimentally-derived measurements.

	Compounds	Lisinopril	Captopril			Spirapril	Lacidipine		Verapamil
Output metrics	ROA	Oral	IV	IV	IV	Oral	Oral	Oral	IV
	Dose, mg	20	2.78	5.67	11.4	25	2	4	5
AUC_0-t_, µg·h/L	Observed	N/A	N/A	N/A	N/A	N/A	3.66	7.66	N/A
	Calculated	752.00	42.97	93.93	215.69	991.83	3.12	6.80	703.03
	Predicted	823.50	49.16	92.55	212.12	977.60	3.27	8.80	494.26
	AAFE	1.10	1.14	1.01	1.02	1.01	1.05	1.29	1.42
	AFE	1.10	1.14	0.99	0.98	0.99	1.05	1.29	0.70
C_max_, µg/L	Observed	N/A	N/A	N/A	N/A	430.00	1.24	3.09	N/A
	Calculated	57.40	104.64	234.55	454.71	378.00	1.17	2.87	1176.82
	Predicted	53.93	105.81	183.46	497.25	196.17	1.00	2.01	1696.96
	AAFE	1.06	1.01	1.28	1.09	1.93	1.16	1.43	1.44
	AFE	0.94	1.01	0.78	1.09	0.52	0.86	0.70	1.44
T_max_, h	Observed	N/A	N/A	N/A	N/A	0.90	1.13	1.25	N/A
	Calculated	6.04	0.15	0.19	0.13	1.00	1.23	1.05	0.09
	Predicted	6.03	0.15	0.19	0.13	1.75	1.05	1.05	0.09
	AAFE	1.00	1.00	1.00	1.00	1.75	1.17	1.00	1.00
	AFE	1.00	1.00	1.00	1.00	1.75	0.86	1.00	1.00
Statistics	Chi-squared	2803.13 *	22.56	23.01	26.85	2.70	107.9 *	182.15 *	11.57 *
	*p*-values	>0.50	>0.50	>0.50	>0.50	<0.001	>0.50	>0.50	0.36

Note: outputs marked as “observed” were extracted directly from the source manuscripts. “Calculated” corresponds to recalculation of the output values using internal non-compartmental methods. Predicted C_max_ values correspond to the maximum sampling concentration within the time range of observed timepoints. For observed datapoints with poorly visible error bars, an effective error was calculated using the variability from other timepoints. Chi-squared values and associated *p*-values were calculated assuming a standard deviation of 0.1× in observed values for manuscript without reported measurement error. * differences considered statistically significant.

**Table 5:** PK metrics calculated from simulations of drug distribution to ACE-expressing organs and tissues.

Metrics	Tissue	Lisinopril, 20 mg, oral	Captopril, 2.78 mg, IV	Spirapril, 25 mg, oral	Lacidipine, 2 mg, oral	Verapamil, 50 mg, IV
AUC_0–t_, µg·h/L	Lung	341.24	52.36	2315.64	13.86	344.12
	Gut	354.03	196.74	2310.31	13.86	354.74
	Nasal tissue	196.91	1.48	207.74	31.63	360.48
	Nasal epithelium	196.34	1.48	207.58	31.63	360.48
C_max_, µg/L	Lung	34.07	15.26	7514.65	140.24	65.26
	Gut	33.68	103.16	3213.00	92.77	70.37
	Nasal tissue	19.67	0.43	657.99	256.36	68.36
	Nasal epithelium	19.67	0.43	478.23	128.78	68.35
T_max_, h	Lung	6.65	1.13	0.08	0.02	1.84
	Gut	5.68	0.57	0.13	0.03	1.30
	Nasal tissue	6.66	1.13	0.09	0.02	1.85
	Nasal epithelium	6.69	1.17	0.11	0.05	1.89

## Data Availability

Data is contained within the article.

## Sample Availability

Samples of the compounds are not available from the authors.
